# Oviposition, Development and Survivorship of the sweetpotato Whitefly *Bemisia tabaci* on Soybean, *Glycine max*, and the Garden Bean, *Phaseolus vulgaris*

**DOI:** 10.1673/031.009.0101

**Published:** 2009-02-04

**Authors:** Augustine Mansaray, Abu James Sundufu

**Affiliations:** ^1^ Institute of Agricultural Research, Njala, Sierra Leone; ^2^ Department of Biological Sciences, School of Environmental Sciences, Njala University, Sierra Leone

**Keywords:** finite rate of growth, net reproductive rate, intrinsic rate of increase

## Abstract

Oviposition, development and survivorship of *Bemisia tabaci* (Gennadius) (Hemiptera: Aleyrodidae) were evaluated on soybean and garden bean under laboratory conditions of 26.0 ± 0.5 °C, 70 – 80% RH and a photoperiod of 14:10 (L:D). *B. tabaci* deposited more eggs and survivorship of nymphs was significantly greater in a choice-test on soybean, *Glycine max* L. (Merr.) (Fabeles: Fabaceae), compared to the garden bean, *Phaseolus vulgaris* L. Overall developmental time from egg to adult eclosion was longer on garden bean than on soybean. Also, *B. tabaci* was more fecund and long-lived on soybean compared to garden bean. Demographic parameters calculated from life tables on the two bean species indicate that soybean is a better host plant for *B. tabaci* than garden bean.

## Introduction

The sweetpotato whitefly, *Bemisia tabaci* (Gennadius) (Hemiptera: Aleyrodidae) biotype B, is currently the most devastating pest in tropical and subtropical countries, due largely to its role in the transmission of a variety of plant viruses (Perring 2001). According to Brown (1994), the distribution of the species is related to intensive agricultural production and the expansion of monoculture practices, associated with indiscriminate use of chemical pesticides. The most outstanding feature of the species is its ability to adapt to a variety of host plants and to unfavourable environmental conditions. Registered hosts include at least 54 plants species from 77 botanical families (Basu 1995). These figures may be underestimated, since non-commercial plant species are seldom included in host range studies.

In addition to direct feeding damage, the insect is the vector of a number of devastating plant viruses, causing debilitating plant disorders of unknown aetiology. By the excretion of honeydew, it reduces the quality of harvested products (Perkins and Bassett 1998; Heinz 1996; Henneberry et al. 1998).

Chemical control is still the key denominator in the management of *B. tabaci*, however, this pest can rapidly develop resistance to insecticides and so the sole reliance on insecticides is unsustainable in the long term (Byrne et al. 2003). Alternative management strategies include natural enemies, including parasitoids and predators, which are regarded as potential agents for use in classical biological control of this pest (Gerling et al. 2001; Ren et al. 2001; Qiu et al. 2005), and host plant resistance. However, management of *B. tabaci* is challenging because of its intercrop movement, high reproductive potential and it's under leaf habitat.

The objectives of this study were to compare *B. tabaci* oviposition and development on soybean, *Glycine max* L. (Merr), and the garden bean, *Phaseolus vulgaris* L. (Fabeles: Fabaceae), evaluate additional life history characteristics and to use this information to suggest ways of integrating host plant resistance, biological control and other non-chemical tactics into management practices for this pest.

## Materials and Methods

The study described was conducted in the Laboratory of Insect Ecology, Department of Entomology, South China Agricultural University, Guangzhou under a mean temperature of 26.0 ± 0.5 °C, 70 to 80% RH, 14:10 L:D photoperiod and a light intensity of 3000 Lux.

### Host plants and whitefly

Seeds of *G. max*, and *P. vulgaris* were obtained from Guangdong Agricultural Institute in Guangzhou, South China. The seeds were placed in Petri dishes with water to initiate germination. The partially germinated seeds were grown individually in 12 cm diameter plastic pots and used in the experiment at the 4–6 leaf stage. These pots were placed into cages (60 × 60 × 60 cm).

*B. tabaci* was originally collected on hibiscus, *Rosa-sinensis* L. (Malvales: Malvaceae) in Teem Plaza, Guangzhou City in 2001, and was identified as B biotype using both RAPDPCR (De Barro and Driver 1997) and mitochondrial COI (Frohlich et al. 1999).

The whitefly was maintained on cucumber, *Cucumis sativus* L. (Cucurbitales: Cucurbitaceae), plants in a greenhouse, and a subcolony maintained in rearing cages (60 × 60 × 60 cm) in the laboratory for two generations before being used in the experiments.

### Feeding and oviposition preference

Choice tests were conducted to compare the feeding and oviposition preference of *B. tabaci* on the two bean species. Six plants of each species were selected. Twenty-four leaves, 12 from each species, were randomly selected and labeled with small pen marks. The plants were individually placed into cages (60 × 60 × 60 cm) into which 240 adult females of *B. tabaci* (10 adult females per leaf) were introduced. These cages were maintained at the controlled conditions described above. The number of *B. tabaci* adults and eggs associated with the leaves of each bean species were recorded after 24 hours. The experiment included six replicates for a total of twelve plants.

### Development and survival of immatures

Small confinement cages were made from transparent reagent bottle cap liners (3 cm diameter and 1.5 cm high), into which a small hole had been punched for ventilation. Approximately 20 pairs of *B. tabaci* adults were released into leaf-clip cages attached to the underside of the leaves of each host plant with the aid of paper clips. Adults were allowed to lay eggs for 12 h before being removed. A small pen mark was used to place identifying marks next to 50 whitefly eggs on each of six leaves per species. The infested plants were placed in 60 × 60 × 60 cm cages and development and survival of each whitefly immature stages on the two bean species were recorded daily until all the whiteflies emerged. With the exception of the crawlers, which are capable of small distance movement immediately after hatching from egg, all the other immature stages are sessile and cannot move. Therefore, leaves with “pupae” were covered with leaf-clip cages to trap emerging adult whiteflies. Emerged adult whiteflies were counted and sexed as described by Gills (1993) and used for daily longevity and fecundity studies.

### Life table

Mated females were obtained and introduced into leaf-clip cages as above. They were then transferred to fresh leaves every 24 hours until death, to determine the daily fecundity (number of eggs laid by female whitefly over her lifetime). Survivorship and number of eggs laid each day were recorded. Fecundity and longevity data were used to calculate daily and lifetime fecundity of *B. tabaci*. Twenty female whiteflies were used for each bean species.

### Statistical analysis

Data for oviposition, feeding preference, developmental time, survival, longevity and fecundity on the two bean species were subjected for analysis of variance, the means were separated using the least significant difference test (LSD) at P < 0.05 (SAS 2001).

A lx-mx life table was constructed for each bean species using sex ratio, survivorship, age-specific fecundity of adults and survivorship and developmental time of all immature stages to calculate intrinsic rate of increase (rm), finite rate of increase (λ), net reproductive rate (Ro), mean generation time (Tc) and doubling time (Td) (Birch 1948).

## Results and Discussion

### Adult feeding and oviposition preference

When given a choice, significantly more *B. tabaci* adult females were found feeding on soybean than garden bean (F = 82.52, df = 1; P = 0.0013) (Table 1). Oviposition was also significantly greater on soybean than on garden bean (F = 59.20, df = 1; P = 0.0001). The preference for soybean over garden bean in terms of both number of whiteflies attracted and oviposition could be possibly due to differences in physical and chemical characteristics of the leaves of the two bean species. In general, hairy plant species have been found to be preferred over globrous ones up to a certain level when hairiness begins to interfere with feeding and attachment of eggs to the leaf epidermis. This premise was supported by Butler and Wilson (1984), who reported that *B. tabaci* showed higher preference for hairy-leaf varieties of cotton to globrous ones. McAuslane et al. (1996) also reported a positive correlation between hairiness and oviposition of *B. tabaci* on soybean. In the present study, *B. tabaci* preferred soybean which has trichomes covering the leaf surface.

**Table 1.**
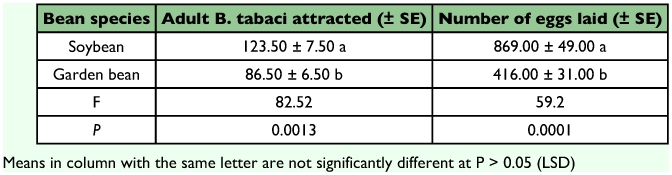
Adult feeding and oviposiiton preferences of Bemisia tabaci on soybean and garden bean

### Immature development and survivorship

*Bemisia tabaci* developed almost 3 days faster on soybean (18.00 ± 0.89 days) than on garden bean (21.19 ± 0.85 days) (F = 33.45, df = 1; P = 0.0045) with the 1st, 2nd, 3rd and 4th instars contributing significantly to the difference (Table 2). The reported mean developmental time for *B. tabaci* on garden bean was close to the value (21.8 days) reported by Eichelkraut and Cardona (1989) under similar conditions of temperature and relative humidity. Generally, developmental time vary greatly with temperature and host plant. However, all the developmental parameters measured in the current study were conducted at 25° C thus; the difference in developmental time between the two bean species could be attributed probably to the plant factor alone. In view of this, Coudried et al. (1985) found that the time required for *B. tabaci* to complete development from egg to adult was influenced by the host plant from which it fed. For instance, mean duration in days varied among hosts: carrot (29.8), broccoli (29.7), tomato (18.6), cotton (21.7), squash (21.3), cucumber (20.6) and sweet potato (18.6). Stage-specific survivorship was also significantly different (P < 0.05) with stages surviving longer on *G. max* than on *P. vulgaris* (Table 3). Survivorship of immatures was significantly (P < 0.05) different on the two bean species with immatures surviving more on *G. max* compared to *P. vulgaris*. Estimates for survivorship of immatures were significantly different, 96.07 % on *G. max* compared to 69.08% on *P. vulgaris* (F = 924.57, df = 1; P = 0.0001) (Table 3). The mechanisms that determine *B. tabaci* choice of a plant as substrate for progeny development have been only partially elucidated. Those include plant colour, texture, free metabolites in the sap, quantity of trichomes in the leaves, and nutritional state, among others (Van Lenteren and Noldus 1990; Bentz et al. 1995; Chu et al. 1995; Andres and Connors 2003). The combination of these factors with abiotic agents for adult dispersal (wind, for example) may determine differential oviposition between plant species in the field (Byrne 1999). Additionally, whiteflies can show some degree of variability in the preference for host plants depending on the time, season, environmental conditions and agronomic practices (Gerling 1990). Considering the short developmental time of *B. tabaci* on *G. max* coupled with the high survivorship of immatures, *B. tabaci* population will build up to a damaging level faster on *G. max* than on *P. vulgaris*. Adult emergence occurred from 16–20 days after oviposition on *G. max* and 19–23 days on *P. vulgaris*.

**Table 2.**
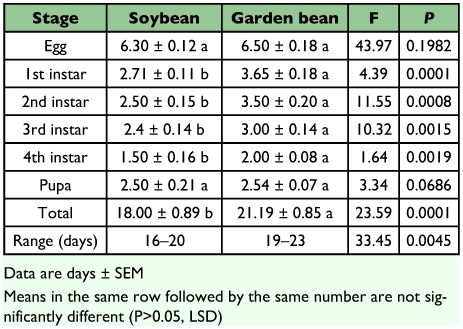
Development of immature stages of Bemisia tabaci on soybean and garden bean reared in the laboratory

### Adult longevity and fecundity

**Figure 1.**
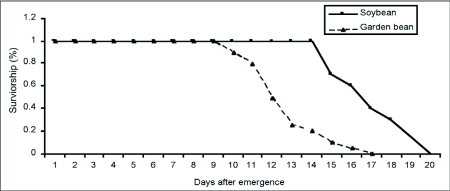
Daily survivorship of *Bemisia tabaci* on *Glycine max* and *Phaseolus vulgaris* in the laboratory (26.0 ± 0.5 °C, 70–80% RH, 14:10 L:D)

Adult longevity was recorded as part of the fecundity experiment. Although most females died by day 13 and 17 on garden bean and *G. max*, respectively, some females were longed lived surviving to 17 days on *P. vulgaris* and 20 days on soybean (Figure 1).

The mean longevities differ significantly (F = 59.22, df = 1; P = 0.0001) between the two experimental plants with females living longer on *G. max* (15.30 ± 4.56 days) than on *P. vulgaris* (10.65 ± 3.25 days) (Table 4). These means were however not too different from the range (10–15 days) reported by Gerling et al. (2001) for *B. tabaci* in the field at temperatures in the higher twenties.

**Table 3.**
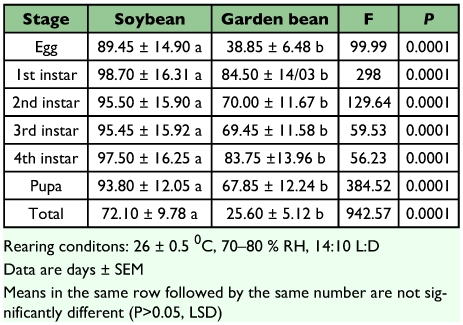
Stage-specific survival of Bemisia tabaci on soybean and garden bean in the laboratory

A preoviposition period of 1day was recorded in the fecundity experiment which is consistent with that reported by Powell and Bellow (1992) for *B. tabaci*. Females laid an average of 163.50 ± 3.91 eggs over their lifetime on *G. max* and 105.35 ± 2.67 eggs on *P. vulgans* (Table 5), these means were significantly different (F = 147.09,df = 1; P = 0.0001). The reason for the difference in fecundity on the two bean species might possibly be attributed to difference in the external physical characteristics of the leaf surface (hairiness) and the internal chemical characteristics of the leaves (pH of leaf sap), with the sap of *G. max* probably being of a better nutritional quality for the whitefly than garden bean. However, daily mean fecundity on the two plants was not significantly different (F = 0.51, df = 1; P = 0.4794).

**Table 4.**
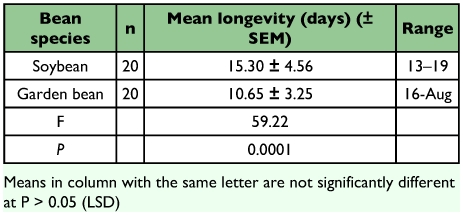
Longevity of Bemisia tabaci on soybean and garden bean

### Life table

Results from the development and fecundity experiments were used to develop lx-mx life tables for *B. tabaci* on the two bean species. These tables were used to calculate demographic parameters shown in Table 6. Sex ratio (female: male) was 1: 0.934 or 51.85% females (n = 150).

The net reproductive rate (Ro) of *B. tabaci* on *G. max* (82.69) was higher than that for *P. vulgaris* (54.98) due to the low survivorship of immatures on the latter. The reproductive rate on *P. vulgaris* was however higher than the value (24.7) reported by Tsai and Wang (1996) for *B. argentifolii* on the same plant. The generation time (Tc) of *B. tabaci* on *G. max* and *P. vulgaris* were 23.89 and 25.92, respectively. The recorded value on *G. max* was similar to but slightly higher than the value (23.2) reported by Tsai and Wang (1996) on cucumber while that on *P. vulgaris* was lower than the value (27.0) reported by the same authors for *B. argentifolii* on *P. vulgaris*, The finite rate of growth (λ) for *G. max* and *P. vulgaris* were (1.69) and (1.87), respectively, while the doubling time (Td) for *G. max* and *P. vulgaris* were (3.85) and (4.48), respectively. The intrinsic rate of natural increase (rm) for *G. max* (0.18) was higher than that for *P. vulgaris* (0.15) probably due to the substantially lower survival rate of immatures on *P. vulgaris.* The recorded rm value on *P. vulgaris* was almost similar to the value (0.153) reported by Tsai and Wang (1996) on tomato for *B. argentifolii*.

**Table 5.**
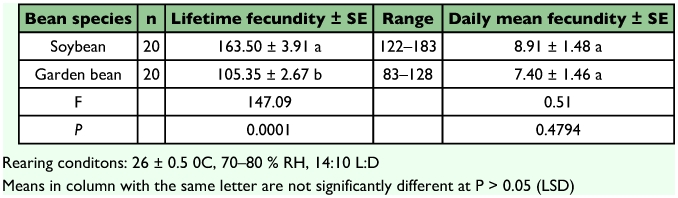
Fecundity of Bemisia tabaci on soybean and garden bean

**Table 6.**
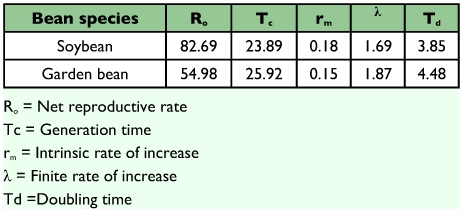
Life table parameters of B. tabaci on soybean and garden bean in a laboratory

Small differences in rm values can make remarkable difference in expected population growth over time. Thus, to compare the population growth of *B. tabaci* on the two host plant over time, the exponential equation for population growth Nt = Noert was used, where No is the initial number of whiteflies on the two plants, Nt is the number of whiteflies at time t, rm is the intrinsic rate of increase and t is the time in days. Given a stable age distribution, the estimated whitefly population on *G. max* with rm (0.18) from a single female will reach 5453.43 within two generations (47.8 days) while on *P. vulgaris* with rm (0.15) and Tc (51.84 days) will only be 2382.72, a 2.26 fold difference. Given these life history parameters, whitefly populations would be expected to build up relatively slowly on *P. vulgaris* than *G. max* and therefore would be easier to manage to a lower population level. The relatively poor host attribute of *P. vulgaris* for *B. tabaci*, causing delayed development, could make it possible for integration with other with other control tactics such as biological control. For instance, the rm values of *Encarsia bimaculata* (Heraty and Polaszek), the principle parasitoid species in Southern China, at 26°C was 0.19 (Qiu et al. 2006), while that of *B. tabaci* B biotype at the same temperature were 0.18 on *G. max* and 0.15 on *P. vulgaris* in the present study. This suggests that at the temperature under consideration, *E bimaculata* could intrinsically control *B. tabaci* B biotype better on *P. vulgaris* than on *G. max*.

Differences in plant infestation will thus be a combination of host preference for oviposition, host suitability for insect development, and the combination effects of natural enemies and other causes of death.
